# Hypothalamic Injury Following Surgery for Craniopharyngioma Causing Immediate Postoperative Death

**DOI:** 10.7759/cureus.18814

**Published:** 2021-10-16

**Authors:** Pooja Bhandari, Sagar Nagpal, Ashwaghosha Parthasarathi, Mohammed M Ahmed, Mayank Jeswani

**Affiliations:** 1 Public Health, Madhavnagar Government Hospital, Ujjain, IND; 2 Internal Medicine, Erie County Medical Center, Buffalo, USA; 3 Internal Medicine, Allergy, Asthma, and Chest Center, Mysore, IND; 4 Surgery, Shyam Shah Medical College, Rewa, IND; 5 Neurosurgery, Gajara Raja Medical College, Gwalior, IND

**Keywords:** left frontotemporal craniotomy, gross total resection (gtr), hypothalamic injury, hypothalamus, craniopharyngioma

## Abstract

While autonomic disturbances resulting from a hypothalamic injury are uncommon complications following surgery for craniopharyngioma, they can lead to postoperative death. Herein, we discuss the case of a multicompartmental craniopharyngioma in a 13-year-old child who died due to unexpected hypothalamic injury, resulting in rapid deterioration in the hemodynamic and neurological status of the patient.

## Introduction

Craniopharyngiomas are rare, benign sellar/parasellar tumors derived from embryonic tissue [1]. Even though these tumors are slow-growing and benign, they cause complications as a result of the mass effect on the neighboring structures. This results in the patient presenting with symptoms, such as headaches, bitemporal hemianopia, and hypopituitarism. 

Radical excision of craniopharyngiomas usually leads to injury to the hypothalamus. However, complications arising from autonomic dysfunction, which was seen in our case, are uncommon and do not respond well to treatment.

## Case presentation

**Figure 1:**
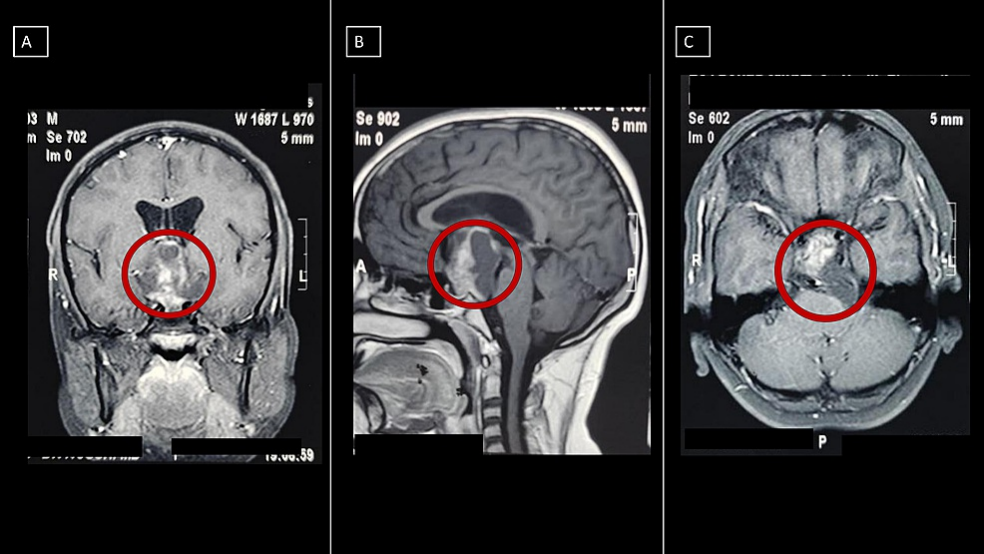
Preoperative contrast-enhanced MRI A: Preoperative contrast-enhanced coronal MRI showing a heterogeneously enhancing lesion in the sellar/suprasellar region; B: preoperative contrast-enhanced sagittal MRI showing the solid cystic nature of the sellar/suprasellar lesion with enhancing solid portion and non-enhancing cystic portion; C: preoperative contrast-enhanced axial MRI showing the solid cystic lesion centred in the sellar/suprasellar region with extension of the cystic component posteriorly into the left cerebellopontine (CP) angle cistern, causing effacement of it and causing mass effect on the left brainstem **The red circle marks the location of the lesion on the MRI

A 13-year-old child was admitted with an eight-month history of headache and recurrent seizures. Neurological examination was unremarkable with no motor or sensory deficits preoperatively. There was, however, bilateral papilledema. MRI was done which revealed that the tumor was multicompartmental, extending from the suprasellar cistern to the right subtemporal region and invading the prepontine cistern, loosely adherent to the basilar artery (Figure 1).

Under the cover of antibiotics, anti-epileptics, and mannitol, a left frontotemporal craniotomy, also known as pterional craniotomy, was done. A transsylvian approach was taken, the arachnoid matter was opened, and the right internal carotid artery and optic nerve were identified. Then, via the optico-carotid cistern, a gross total resection (GTR) of the tumor was performed with sufficiently safe margins to avoid the risk of recurrence. Except for the hypothalamus, there was a good cleavage plane from the surrounding neural and vascular structures and the tumor.

The whole operative procedure was uneventful. The patient was hemodynamically stable with a normal sensorium immediately after surgery. The only abnormalities noticed were right lateral rectus weakness concomitant with subtle right-sided pyramidal weakness. 

On the fifth day, he developed diabetes insipidus with a serum sodium concentration of 165 mEq/L and an increased urine output of approximately 2.5 L - 3 L per day. He was treated with desmopressin spray and intravenous fluids. On the evening of postoperative Day 7, the patient started developing hypothermia with a temperature of 33°C and started shivering. Arterial blood gas (ABG) analysis was done frequently and was found to be normal. A postoperative CT scan of the brain showed a left frontotemporal craniotomy defect with postoperative changes at the sellar and suprasellar region and minimal residual tumor with perilesional edema.

The next morning, he was found to be in a Glasgow Coma Scale (GCS) of 7 which required immediate intubation and ventilation. The next day, the patient developed wide blood pressure ﬂuctuations. An echocardiogram identified a left ventricular dysfunction. Soon after, despite the resuscitative measures, the patient succumbed to a fatal cardiac arrest. 

## Discussion

Despite all of the recent advances in operative techniques and surgical equipment, craniopharyngioma is considered difficult to treat; the optimal treatment strategy for it remains ambiguous [2].

A craniopharyngioma is a benign tumor, GTR is considered to be the gold standard. However, this approach can be challenging due to the location of these tumors, which is usually in close proximation with structures, such as the optic chiasm, hypothalamus, and internal carotid arteries [3-4]. A more conservative approach is a subtotal resection (SR) with subsequent radiation therapy (RT) which consists of deliberately leaving around 10% of the residual lesion. This approach has been shown to have reduced surgical complications, but there are higher chances of tumor recurrence and adverse effects from radiation [5-6]. 

**Figure 2:**
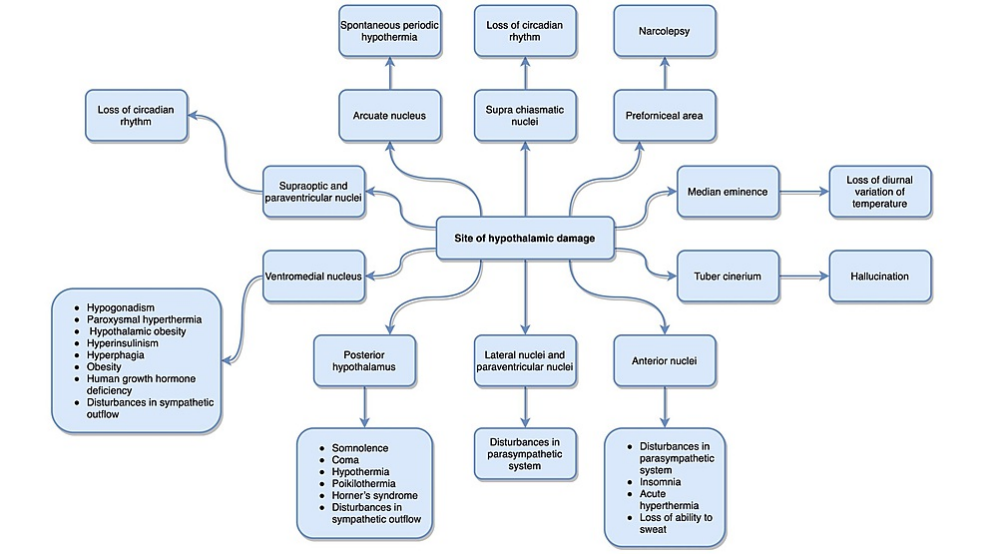
Flowchart illustrating clinical manifestations of destruction of various regions of the hypothalamus

Postoperative morbidities due to GTR commonly encompass endocrinopathies leading to panhypopituitarism; however, death as a result of hypothalamic damage is rarely reported [2, 7]. Clinical manifestations due to the destruction of various regions of the hypothalamus are illustrated in Figure 2.

Based on the clinical characteristics of the patient, it can be hypothesized that the following areas of the hypothalamus were damaged: supraoptic and paraventricular nuclei (causing diabetes insipidus) as well as the posterior hypothalamus, ventromedial, lateral, paraventricular, and anterior nuclei (causing hypothermia, tachycardia, and wide ﬂuctuations in blood pressure). The cardiac dysfunction was a result of the injury to the autonomic region of the hypothalamus resulting in ventricular failure and eventually death. 

It is our belief that our attempt at GTR proved to be fatal due to the damage to multiple hypothalamic nuclei. This injury to the hypothalamus may have been a result of direct trauma to it or an infarction following injury to the vessels that supply the hypothalamus. A less radical approach, such as SR with RT, would have probably avoided the patient’s mortality, especially when we noticed multiple hypothalamic nuclei adherent to the surface of the tumor. This is further substantiated by a systematic review done by Yang et al. that showed that SR + RT was an acceptable approach to achieve tumor control while limiting hypothalamic morbidity associated with GTR [8].

## Conclusions

GTR is considered to be the gold standard treatment for craniopharyngioma and is successful in most cases as it hinders the chances of recurrences of the tumor. However, in our case, the tumor was noted to be multi-compartmental and was adherent to multiple nuclei of the hypothalamus. The use of GTR in such circumstances led to damage of various nuclei of the hypothalamus, resulting in deterioration in the patient’s health and, finally, led to his demise. We conclude that, in cases such as ours, the idea of SR followed by RT may prove to be a better approach despite having a higher recurrence rate because the life-threatening extreme damage to the hypothalamic nuclei can be avoided.
